# The Contributions of Selected Diseases to Disparities in Death Rates and Years of Life Lost for Racial/Ethnic Minorities in the United States, 1999–2010

**DOI:** 10.5888/pcd11.140138

**Published:** 2014-07-31

**Authors:** George Howard, Frederick Peace, Virginia J. Howard

**Affiliations:** Author Affiliations: Frederick Peace, Virginia J. Howard, School of Public Health, University of Alabama at Birmingham, Birmingham, Alabama.

## Abstract

**Introduction:**

Differences in risk for death from diseases and other causes among racial/ethnic groups likely contributed to the limited improvement in the state of health in the United States in the last few decades. The objective of this study was to identify causes of death that are the largest contributors to health disparities among racial/ethnic groups.

**Methods:**

Using data from WONDER system, we measured the relative (age-adjusted mortality ratio [AAMR]) and absolute (difference in years of life lost [dYLL]) differences in mortality risk between the non-Hispanic white population and the black, Hispanic, American Indian/Alaska Native, and Asian/Pacific Islander populations for the 25 leading causes of death.

**Results:**

Many causes contributed to disparities between non-Hispanic whites and blacks, led by assault (AAMR, 7.56; dYLL, 4.5 million). Malignant neoplasms were the second largest absolute contributor (dYLL, 3.8 million) to black–white disparities; we also found substantial relative and absolute differences for several cardiovascular diseases. Only assault, diabetes, and diseases of the liver contributed substantially to disparities between non-Hispanic whites and Hispanics (AAMR ≥ 1.65; dYLL ≥ 325,000). Many causes of death, led by assault (AAMR, 3.25; dYLL, 98,000), contributed to disparities between non-Hispanic whites and American Indians/Alaska Natives; Asian/Pacific Islanders did not have a higher risk than non-Hispanic whites for death from any disease.

**Conclusion:**

Assault was a substantial contributor to disparities in mortality among non-Asian racial/ethnic minority populations. Research and intervention resources need to target diseases (such as diabetes and diseases of the liver) that affect certain racial/ethnic populations.

## Introduction

A recent report underscored dramatic improvements in the state of health in the United States but also noted that improvements have not been as rapid as they have been in other wealthy nations (1). Differential risk in subpopulations (health disparities) is a likely contributor to this shortfall. Life expectancy differs among racial/ethnic groups; in 2006, life expectancy for whites was 78.5 years, lower than the life expectancy for Asians (86.6 y) or Hispanics (82.8 y) but higher than the life expectancy for blacks (73.4 y) or American Indians or Alaska Natives (74.2 y) (2). However, differences in life expectancy do not account for racial/ethnic differences in causes of death.

The Minority Health and Health Disparities Research and Education Act of 2000 requires the National Institutes of Health to study health disparities among racial/ethnic groups. However, the law does not provide guidance for allocating resources for research on specific diseases or racial/ethnic groups. One study noted a confounding of race and region, with different life expectancies for whites in Appalachia and whites in the Mississippi Valley, low-income rural whites in the northern plains and the Dakotas, and whites from other regions; and different life expectancies for southern low-income rural blacks, high-risk urban blacks, and other blacks (3). 

Because of limited funding to investigate disparities, resources should be allocated according to the impact of the disparity. Should this allocation be driven by data on relative differences or data on absolute differences? Suppose 2 diseases are competing for resources. The first disease demonstrates a large relative difference in mortality risk among racial/ethnic groups; however, the disease is rare, or the racial/ethnic group affected is small. The second disease demonstrates a small relative difference in mortality risk among racial/ethnic groups, but the disease is common, and the racial/ethnic group affected is large, so the disease and the disparity affect many people. There is value in addressing both large relative and large absolute differences in mortality risk. The objective of this study was to provide measures of relative and absolute differences in mortality risk for the 25 leading causes of death in 5 racial/ethnic groups: American Indians and Alaska Natives (AIAN), Asians and Pacific Islanders (API), blacks, Hispanics, and non-Hispanic whites.

## Methods

Data were retrieved from the Wide-Ranging Online Data for Epidemiologic Research (WONDER) system supported by the Centers for Disease Control and Prevention (4). In 1999, the WONDER system converted codes for cause of death to *International Classification of Diseases, 10th Revision* (ICD-10) codes; data are available for a 12-year period through 2010. For each ICD-10 subchapter, we first retrieved data on the number of deaths and calculated the total number of person-years at risk by summing the population in each of the 12 study years. We then tabulated age-adjusted (to the 2000 US population) mortality rates for each racial/ethnic group (non-Hispanic white, black, Hispanic, American Indian/Alaska Native [AIAN], Asian/Pacific Islander [API]). Because the number of deaths was too small to provide stable estimates, our racial/ethnic classification excluded a small number of Hispanic AIAN, Hispanic API, and Hispanic blacks from the analyses (collectively 4,072,827 of 3,530,708,204 [0.1%] of the total person-years at risk).

The relative measure of disparity was the age-adjusted mortality ratio (AAMR), calculated as the age-adjusted mortality rate for the racial/ethnic minority group of interest divided by the age-adjusted mortality rate for non-Hispanic whites. The absolute measure of disparity was difference in years of life lost (dYLL), derived by first calculating the “excess” (or “deficit”) number of deaths in each age stratum (<1 y, 1–4 y, 5–9 y, 10–14 y, 15–19 y, 20–24 y, 25–34 y, 35–44 y, 45–54 y, 55–64 y, 65–74 y, 75–84, and ≥85 y) as the difference between the observed number of deaths and the expected number of deaths (population of racial/ethnic minority group × age stratum death rate for non-Hispanic whites) for the stratum. For each age stratum, the sum of the expected years of life was calculated from the non-Hispanic white life table (5), and the dYLL calculated as the sum of the years of life lost across the age strata. 

In our initial analysis, the top 25 causes of death included only 86% of the deaths for the black and Hispanic populations and 87% for the AIAN population; this raised the possibility that other diseases could be important in these populations. A post hoc analysis examined the next 25 leading causes of death. 

## Results

Overall, the 25 leading causes of death caused 90% of all deaths (Table 1). Malignant neoplasms ranked first as cause of death among all racial/ethnic groups, according to age-adjusted mortality rates (Table 2).

**Table 1:** No. (%) of People Who Died of Any of the 25 Leading Causes of Death^a^, by Racial/Ethnic Group^b^, United States, 1999–2010

Category	Total	Racial/Ethnic Group				
		Non-Hispanic White	Non-Hispanic Black	Hispanic White	Non-Hispanic American Indian or Alaska Native	Non-Hispanic Asian or Pacific Islander
**Person-years at risk^c^**	3,486,559,143	2,384,810,893	445,142,145	463,832,712	29,273,028	163,500,365
**ICD-10 subchapter group**						
C00–C97: Malignant neoplasms	6,694,576 (23)	5,484,107(23)	751,193 (22)	298,644 (20)	27,579 (18)	133,053 (27)
I20–I25: Ischemic heart diseases	5,400,064 (19)	4,503,062 (19)	548,407 (16)	241,062 (16)	20,851 (13)	86,682 (17)
I30–I51: Other forms of heart disease	1,878,942 (6)	1,573,578 (7)	211,619 (6)	62,144 (4)	7,105 (5)	24,496 (5)
I60–I69: Cerebrovascular diseases	1,772,800 (6)	1,434,266 (6)	211,205 (6)	78,900 (5)	6,538 (4)	41,891 (8)
J40–J47: Chronic lower respiratory diseases	1,538,164 (5)	1,382,607 (6)	94,921 (3)	39,317 (3)	6,128 (4)	15,191 (3)
E10–E14: Diabetes mellitus	853,442 (3)	607,583 (3)	147,577 (4)	71,592 (5)	8,808 (6)	17,882 (4)
G30–G31: Other degenerative diseases of the nervous system	826,704 (3)	740,689 (3)	49,980 (1)	26,741 (2)	1,829 (1)	7,465 (2)
W00–X59: Other external causes of accidental injury	783,996 (3)	627,772 (3)	82,237 (2)	54,955 (4)	8,204 (5)	10,828 (2)
F01–F09: Organic, including symptomatic, mental disorders	759,587 (3)	667,749 (3)	58,872 (2)	22,367 (2)	1,833 (1)	8,766 (2)
J09–J18: Influenza and pneumonia	711,031 (2)	591,688 (3)	66,133 (2)	34,193 (2)	3,777 (2)	15,240 (3)
I10–I15: Hypertensive diseases	646,393 (2)	451,799 (2)	144,797 (4)	33,624 (2)	2,501 (2)	13,672 (3)
V01–V99: Transport accidents	536,681 (2)	384,677 (2)	65,348 (2)	65,716 (4)	9,482 (6)	11,458 (2)
N17–N19: Renal failure	493,520 (2)	365,965 (2)	90,113 (3)	26,050 (2)	2,909 (2)	8,483(2)
K70–K76: Diseases of liver	445,324 (2)	331,776 (1)	44,750 (1)	53,549 (4)	8,902 (6)	6,347 (1)
A30–A49: Other bacterial diseases	424,411 (1)	319,234 (1)	75,627 (2)	20,828 (1)	2,609 (2)	6,113 (1)
I70–I78: Diseases of arteries, arterioles, and capillaries	408,985 (1)	348,736 (1)	38,745 (1)	14,061 (1)	1,439 (1)	6,004 (1)
X60–X84: Intentional self–harm	393,603 (1)	331,714 (1)	23,433 (1)	25,101 (2)	4,219 (3)	9,136 (2)
K55–K63: Other diseases of intestines	251,124 (1)	211,341 (1)	24,955 (1)	10,538 (1)	1,321 (1)	2,969 (1)
J60–J70: Lung diseases due to external agents	214,234 (1)	185,016 (1)	18,528 (1)	6,919 (0)	851 (1)	2,920 (1)
G20–G25: Extrapyramidal and movement disorders	225,214 (1)	205,434 (1)	8,079 (0)	7,761 (1)	633 (0)	3,307 (1)
X85–Y09: Assault	204,201 (1)	64,483 (0)	96,072 (3)	36,787 (2)	2,654 (2)	4,205 (1)
J80–J84: Other respiratory diseases principally affecting the interstitium	199,234 (1)	166,500 (1)	14,049 (0)	13,247 (1)	1,524 (1)	3,914 (1)
E70–E88: Metabolic disorders	198,649 (1)	165,623 (1)	21,016 (1)	8,090 (1)	884 (1)	3,036 (1)
R95–R99: Ill–defined and unknown causes of mortality	197,958 (1)	143,082 (1)	34,611 (1)	15,639 (1)	1,923 (1)	2,703 (1)
N30–N39: Other diseases of urinary system	165,358 (1)	137,866 (1)	18,141 (1)	6,491 (0)	677 (0)	2,183 (0)
**All other causes**	2,840,148 (10)	2,091,320 (9)	481,042 (14)	199,533 (14)	19,722 (13)	48,531 (10)

Abbreviations: ICD-10, *International Classification of Diseases, 10th Revision.*

a Source: Wide-Ranging Online Data for Epidemiologic Research (WONDER) system supported by the Centers for Disease Control and Prevention (4). Categories of causes of death established by the ICD-10.

b Because the number of deaths was too small to provide stable estimates, our racial/ethnic classification excluded a small number of Hispanic American Indians/Alaska Natives, Hispanic Asians/Pacific Islanders, and Hispanic blacks from the analyses (collectively 4,072,827 of 3,530,708,204 [0.1%] of the total person-years at risk).

c Sum of the population in each of the 12 years of study.

**Table 2:** Age-Adjusted Mortality Rates for the 25 Leading Causes Of Death^a^, by Racial/Ethnic Group^b^, United States, 1999–2010

ICD-10 Subchapter Group^c^	Age-Adjusted Mortality Rate				
	Non-Hispanic White	Non-Hispanic Black	Hispanic White	Non-Hispanic American Indian or Alaska Native	Non-Hispanic Asian or Pacific Islander
C00–C97: Malignant neoplasms	188.51	229.39	131.72	150.97	113.41
I20–I25: Ischemic heart diseases	151.27	179.46	127.12	127.30	86.67
I30–I51: Other forms of heart disease	52.77	67.22	31.27	43.67	24.70
I60–I69: Cerebrovascular diseases	47.96	69.28	40.28	42.61	41.54
J40–J47: Chronic lower respiratory diseases	46.89	30.70	21.32	38.37	15.87
E10–E14: Diabetes mellitus	20.78	46.38	34.19	49.19	16.95
G30–G31: Other degenerative diseases of the nervous system	24.30	18.93	16.80	15.00	8.79
W00–X59: Other external causes of accidental injury	23.66	21.35	16.25	33.81	9.02
F01–F09: Organic, including symptomatic, mental disorders	21.77	22.27	14.26	15.41	10.55
J09–J18: Influenza and pneumonia	19.75	22.06	18.13	24.53	16.59
I10–I15: Hypertensive diseases	15.15	45.27	16.76	14.92	13.95
V01–V99: Transport accidents	15.74	15.10	14.81	32.99	7.46
N17–N19: Renal failure	12.32	28.99	12.84	17.28	8.40
K70–K76: Diseases of liver	11.81	11.73	19.45	36.09	4.96
A30–A49: Other bacterial diseases	10.90	24.03	9.58	14.30	5.79
I70–I78: Diseases of arteries, arterioles and capillaries	11.71	12.88	7.36	9.36	5.98
X60–X84: Intentional self–harm	13.22	5.39	6.06	14.15	5.68
K55–K63: Other diseases of intestines	7.12	8.16	5.37	8.19	3.00
G20–G25: Extrapyramidal and movement disorders	6.89	2.93	4.56	4.77	3.61
J60–J70: Lung diseases due to external agents	6.16	6.45	3.90	5.89	3.29
X85–Y09: Assault	2.73	20.63	7.22	8.87	2.47
R95–R99: Ill–defined and unknown causes of mortality	5.55	8.35	3.87	7.20	1.92
J80–J84: Other respiratory diseases principally affecting the interstitium	5.68	4.21	6.46	9.25	3.77
E70–E88: Metabolic disorders	5.70	6.79	3.66	5.04	2.90
N30–N39: Other diseases of urinary system	4.57	6.48	3.70	4.79	2.39

Abbreviations: ICD-10, *International Classification of Diseases, 10th Revision.*

a Source: Wide-Ranging Online Data for Epidemiologic Research (WONDER) system supported by the Centers for Disease Control and Prevention (4). Categories of causes of death established by the ICD-10.

b Because the number of deaths was too small to provide stable estimates, our racial/ethnic classification excluded a small number of Hispanic American Indians/Alaska Natives, Hispanic Asians/Pacific Islanders, and Hispanic blacks from the analyses (collectively 4,072,827 of 3,530,708,204 [0.1%] of the total person-years at risk).

c Ranked in decreasing order by death rate for the non–Hispanic white population.

For the black population, the largest AAMR (7.56) and largest dYLL (4.5 million) was for assault (Figure, Panel A, and Supplemental Table 1 in the Appendix). Because malignant neoplasm was a common cause of death, it contributed to the second largest dYLL (nearly 3.8 million) despite a small AAMR (1.22). Similarly, ischemic heart disease (AAMR, 1.19; dYLL, 2.7 million), other forms of heart disease (AAMR, 1.27; dYLL, 2.0 million), and cerebrovascular disease (AAMR, 1.44; dYLL, 2.0 million) had modest AAMRs but contributed to large dYLLs. In contrast, hypertensive diseases (AAMR, 2.99; dYLL, 2.3 million), diabetes (AAMR, 2.23; dYLL, 1.8 million), renal failure (AAMR, 2.35; dYLL, 1.2 million), and other bacterial diseases (AAMR, 2.20; dYLL, 980,000) were less common causes of death, but their higher relative risk contributed to large dYLLs.

**Figure:**
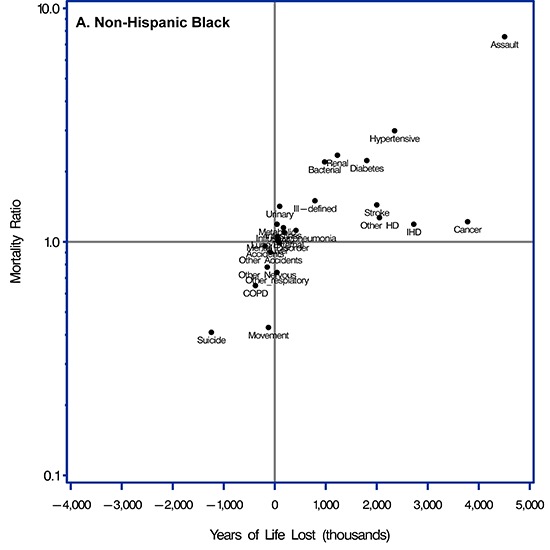

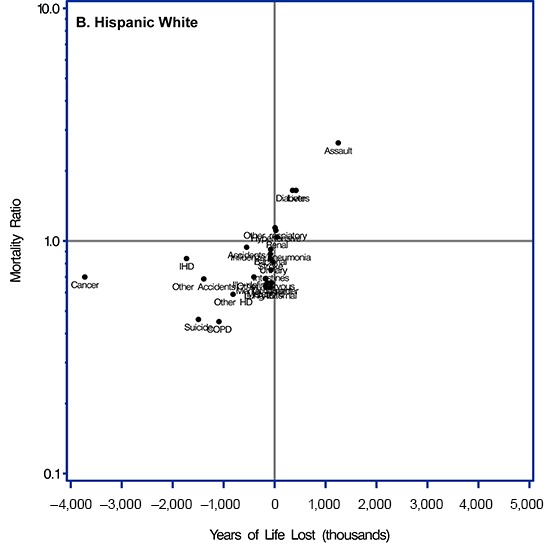

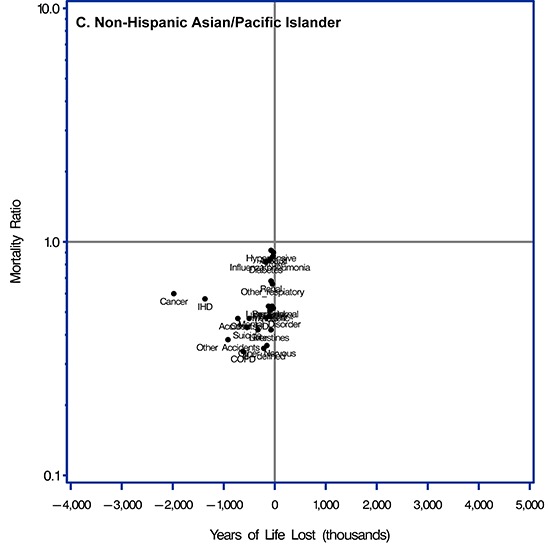

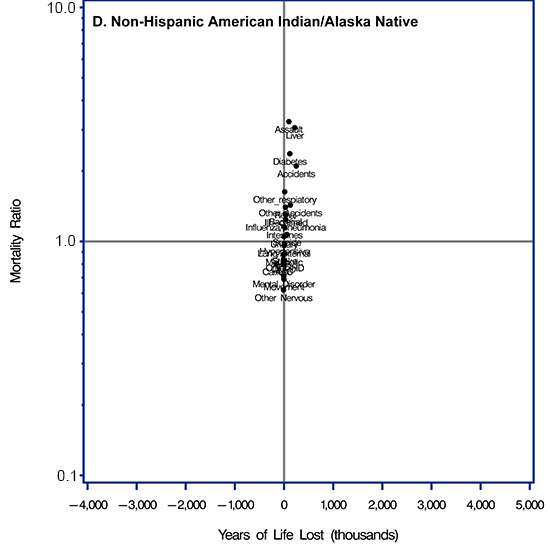
Scatterplots of relative measure of disparities (age-adjusted mortality ratio [AAMR]) and absolute measures of disparities (difference in years of life lost [dYLL]) for each of 4 racial/ethnic minority groups compared with the non-Hispanic white population for the 25 leading causes of death in the United States, 1999–2010. Causes of death were categorized according to the *International Classification of Diseases, 10^th^ Revision*. Scatterplot A is for non-Hispanic blacks; B for Hispanic whites; C for non-Hispanic Asians/Pacific Islanders; and D for non-Hispanic American Indians/Alaska Natives. Abbreviations for categories of causes of death were used to provide clearer plots. Abbreviations: Cancer, malignant neoplasms; IHD, ischemic heart disease; Other HD, other forms of heart disease; Stroke, cerebrovascular disorders; COPD, chronic lower respiratory diseases; Diabetes, diabetes mellitus; Other Nervous, other degenerative diseases of the nervous system; Other Accidents, other external causes of accidental injury; Mental Disorder, organic, including symptomatic, mental disorders; Influenza/Pneumonia, influenza or pneumonia; Hypertensive, hypertensive diseases; Transport, transport accidents; Renal, renal failure; Liver, diseases of the liver; Bacterial, other bacterial diseases; AAC, diseases of the arteries, arterioles or capillaries; Suicide, intentional self-harm; Intestines, other diseases of the intestines; Movement, extrapyramidal or movement disorders; Lung/External, lung diseases due to external agents; Assault, assault; Ill-Defined, ill-defined or unknown causes of mortality; Other Respiratory, other respiratory diseases principally affecting the interstitium; Metabolic, metabolic disorders; Urinary, other diseases of the urinary systems.

Blacks had a lower risk for death from several causes than did whites. Among those causes, the largest dYLL between blacks and whites was for intentional self-harm (AAMR, 0.41; dYLL = −1.2 million). Blacks had a lower relative risk for death from extrapyramidal or movement disorders (AAMR, 0.43; dYLL = −123,000), chronic lower respiratory diseases (AAMR, 0.65; dYLL, −379,000), other respiratory diseases principally affecting the interstitium (AAMR, 0.74; dYLL, −44,000), and other degenerative diseases of the nervous system (AAMR, 0.78; dYLL, −147,000), but these diseases generally contributed to small dYLLs.

Assault was the largest relative and absolute contributor to disparities between whites and Hispanics (AAMR, 2.64; dYLL, 1.2 million) (Figure, Panel B, and Supplemental Table 1). However, the Hispanic population had a substantially higher risk for death from only 2 other causes: diseases of the liver (AAMR, 1.65; dYLL, 421,000) and diabetes (AAMR, 1.65; dYLL, 353,000). The Hispanic population had a lower risk than whites for death from malignant neoplasms (AAMR, 0.70; dYLL, −3.7 million) and ischemic heart disease (AAMR, 0.84; dYLL, −1.7 million), and both common diseases contributed to large dYLLs. Hispanics had substantially lower risk for death from intentional self-harm (AAMR, 0.47; dYLL, −1.5 million), chronic lower respiratory diseases (AAMR = 0.45; dYLL, −1.1 million), other external causes of accidental injury (AAMR, 0.69; dYLL, −1.4 million), and other forms of heart diseases (AAMR, 0.59; dYLL, −814,000), and these substantially lower relative risks contributed to large dYLLs.

The AIAN population had a higher risk than the white population for death from several causes (Figure, Panel C, and Supplemental Table 1); however, the smaller size of the AIAN population contributed to modest dYLLs. Higher relative risks for death from assault (AAMR, 3.25; dYLL, 98,000) and diseases of the liver (AAMR, 3.06; dYLL, 218,000) were striking, but they contributed to small dYLLs; differences in risk for death from diabetes (AAMR, 2.37; dYLL, 118,000) and transportation accidents (AAMR, 2.10; dYLL, 248,000) also contributed to small dYLLs. Many other diseases posed a higher risk for the AIAN population than for whites, including other respiratory diseases principally affecting the interstitium (AAMR,1.64; dYLL, 14,000), renal failure (AAMR, 1.40; dYLL, 28,000), other bacterial diseases (AAMR, 1.31; dYLL, 28,000), influenza and pneumonia (AAMR, 1.24; dYLL, 34,000), ill-defined or unknown causes of mortality (AAMR, 1.30; dYLL, 40,000), and other diseases of the intestine (AAMR, 1.15; dYLL, 8,000).

The AIAN population had a lower risk for death than the white population from several diseases, including other degenerative diseases of the nervous system (AAMR, 0.62; dYLL, −11,000), extrapyramidal and movement disorders (AAMR, 0.69; dYLL, −3,000), and organic mental disorders (AAMR, 0.71, dYLL, −7,000). In addition, the AIAN population had a lower relative risk for all 5 leading causes of death: malignant neoplasms (AAMR, 0.80; dYLL, −158), ischemic heart diseases (AAMR = 0.84; dYLL, −12,000), other forms of heart disease (AAMR, 0.83; dYLL, 14,000), cerebrovascular disease (AAMR, 0.89; dYLL, 13K), and chronic lower respiratory diseases (AAMR, 0.82; dYLL, −14,000).

Finally, the API population had neither a higher relative risk nor a higher absolute risk than the white population for any cause of death, but it did have substantially fewer years of life lost and a lower risk for death from malignant neoplasms (AAMR, 0.60; dYLL, −2.0 million) and ischemic heart disease (AAMR, 0.57; dYLL, −1.4 million) (Figure, Panel D, and Supplemental Table 1).

The post hoc analysis of the 26th- to 50th-ranked causes of death (Supplemental scatterplots in Appendix and Supplemental Table 2) showed a pattern similar to that for the leading 25 causes of death. Numerous diseases contributed to disparities between whites and blacks and between whites and the AIAN population, whereas few diseases contributed to disparities between whites and Hispanics and between whites and the API population. Human immunodeficiency virus (HIV) was the 29th leading cause of death overall; for blacks, the AAMR was a remarkable 10.8 (dYLL, 2.9 million), placing HIV among the major contributors to disparities in mortality for blacks. HIV also played a substantial role in disparities for the Hispanic (AAMR, 2.80; dYLL, 449,000) and AIAN (AAMR, 1.60; dYLL, 12,000) populations. Childhood diseases were particularly noteworthy contributors to disparities between whites and blacks, especially disorders related to the length of gestation and fetal growth (AAMR, 3.79; dYLL, 1.3 million) and respiratory and cardiovascular disorders specific to the perinatal period (AAMR, 2.59; dYLL, 648,000). For Hispanics, only viral hepatitis (AAMR, 3.65; dYLL, 124,000) was a striking contributor to disparities. For the AIAN population, substantial contributors to disparities were substance abuse (AAMR, 3.27; dYLL, 2,000), viral hepatitis (AAMR, 1.82; dYLL, 63,000) and systemic connective disease disorders (AAMR, 1.62; dYLL, 36,000). The dearth of diseases contributing to disparities between whites and the API population remained in the post hoc analysis.

## Discussion

A few causes of death had a similar effect across racial/ethnic groups, whereas many others had different effects for different races or ethnicities. Assault played a major role in deaths among the black, Hispanic, and AIAN populations. Diabetes and diseases of the liver were the only other substantial contributors to relative or absolute disparities for the Hispanic population and were also the second- and third-largest contributors to disparities for the AIAN population. In contrast, the 4 leading causes of death (malignant neoplasms, ischemic heart disease, other forms of heart disease, and cerebrovascular disorders) placed blacks at higher risk than whites but placed all other racial/ethnic minority groups at lower risk. Hypertensive diseases, renal diseases, and other bacterial diseases increased disparities for the black population only. Hence, broad prevention strategies related to assault, diseases of the liver, and diabetes could reduce disparities in many racial/ethnic groups; our data suggest the need for targeting resources to certain combinations of causes of death that affect certain racial/ethnic populations more than others.

Compared with whites, the black and AIAN populations had a higher risk for death from numerous causes, while the Hispanic population had a higher risk from only 3 causes (assault, diseases of the liver, and diabetes), and the API population did not have a higher risk than whites from any cause. After assault, the largest dYLLs in any racial/ethnic group resulted from 3 diseases in the black population: malignant neoplasms, ischemic heart disease, and other forms of heart disease; each disease contributed to more than 2.0 million dYLLs. However, none of these 3 diseases contributed to disparities in the Hispanic or API population. Importantly, compared with the Hispanic population and the API population, the white population had a higher risk of death from cancer, ischemic heart disease, and other forms of heart disease. Cerebrovascular disease contributed to a substantial dYLL for the black population but had almost no effect on dYLLs for other racial/ethnic groups. These patterns are reflected in patterns of life expectancy (2), with shorter life expectancies for the black and AIAN populations and longer life expectancies for the Hispanic and API populations than for the white population.

Focusing on relative disparities shifts attention to several additional diseases: risk was more than double for the black population than for the white population for hypertensive diseases (AAMR, 2.99), renal failure (AAMR, 2.35) and other bacterial diseases (AAMR, 2.20); no other disease approached a doubling of risk for any racial/ethnic group.

If diseases related to vascular diseases and its risk factors were clustered (ischemic heart disease, other forms of heart disease, cerebrovascular diseases, diabetes, hypertension, and renal disease), they would contribute a remarkable 12.1 million in dYLL and dominate all other contributors to disparities between blacks and whites (and have little impact on other racial/ethnic groups).

With the goal of improving the health of people in all racial/ethnic groups in the United States, one could also attempt to find the largest disparities for whites by combining data for all other racial/ethnic groups. When we used this approach, we found that whites had 3.2 million in dYLL from intentional self-harm, 2.3 million in dYLL from other external causes of accidental injury, 2.1 million in dYLL from chronic lower respiratory diseases, 2.1 million in dYLL from cancers, and 1.2 million in dYLL from transportation accidents. There seems to be a promising opportunity to study these causes of death that lead to lower life expectancy among whites.

The objective of this paper was to identify the causes of death that contribute the most to relative and absolute measures of disparities in mortality among racial/ethnic groups and thereby provide a framework to guide investigators to efficiently investigate the contributors to (or mechanisms for) these disparities. These contributors and mechanisms will likely differ among causes and racial/ethnic groups because of the heterogeneous mixture of individual characteristics (socioeconomic, psychosocial, genetic sources, and physiological) and societal exposures (availability of medical resources, neighborhood characteristics, environmental exposures, and others). Consider, for example, disparities in assault and diabetes. It seems likely that disparities in assault would be tightly tied to socioeconomic and psychosocial sources, whereas differences in diabetes would be driven more by physiological or genetic differences. However, by focusing on race–disease combinations that evidence the greatest disparities, further work can increase understanding of the contribution of specific diseases, and interventions can be developed to reduce these disparities. To make this task even more challenging, there are likely dynamic changes in the impact of diseases as contributors to disparities in mortality associated with shifts in deaths from certain diseases (1) and temporal changes in certain diseases that differ between men and women (6) and between regions of the country (7).

This study has several limitations. The estimates from the WONDER system rely on data from death certificates, introducing 2 concerns. The first is misclassification of race/ethnicity: as noted by Arias and colleagues (8), the numerator of the death rate (the number of deaths) is based on the race/ethnicity indicated on the death certificate, information usually collected by physicians or funeral directors who gather the information from personal observation or from next of kin. The denominator of the death rate, the population size, is based on self-reported race/ethnicity from the US Census. That these data come from different sources introduces the possibility of differential reporting by racial/ethnic group and may affect the magnitude of disparity among racial/ethnic groups. Arias and colleagues concluded that this concern is minimal for data on whites and blacks and modest for Hispanic and API populations but may be substantial for AIAN populations. The second concern is the misclassification of cause of death. One study (9) examined data on underlying cause of death obtained from physician-adjudicated medical records (the gold standard) and compared these data with the underlying cause of death indicated on the death certificate. Considering cause of death on a broad basis, the study found that sensitivity ranged from 31% (death from infection) to 81% (death from cancer) and that the positive predictive value ranged from 31% (death from infection) to 50% (death from cancer); however, these gaps in the ability to identify diseases on death certificates would be smaller if a finer classification of causes of death existed (eg, an exact cause instead of a broad classification, such as cancer) Hence, misclassification could play a potentially different role across diseases.

Racial/ethnic differences in health exist in many domains that do not result in death and are not identified in these analyses. For example, mental health problems and diabetes have a profound impact on disparities in quality of life that are identified by measures such as the disability-adjusted life year (DALY) or quality-adjusted life year (QALY) but not by measures of mortality. However, it could be argued that events tied to death are the most serious and important and perhaps should be a primary guide to understanding disparities. In addition, several causes of death share risk factors and are correlated. A competing-cause analysis would partially address this question but is not feasible because of the number of causes of death that need to be considered.

In conclusion, although we found similarities in the contribution of causes of death to racial/ethnic disparities, we found larger differences. The importance of finding the contributors to disparities is growing, and without targeted studies the magnitude of disparities will move from bad to worse. For example, despite the remarkable and rapid 37% decline in stroke mortality (from 173.2 per 100,000 in 1999 to 108.6 per 100,000 in 2009), the black–white stroke disparity increased by 25% (from a mortality ratio of 1.36 in 1999 to 1.45 in 2009 [10]). Not only are there apparent increases in the geographic disparities in life expectancy (7) but disparities associated with socioeconomic status are likely growing; for example, wealthy counties have larger declines in cigarette smoking than poor counties (11). We hope that our data can be used as a road map to guide a call-to-action to aggressively target the contributors to racial/ethnic disparities and lead to interventions that will reduce them.

## Figures and Tables

**Supplemental Figure:**
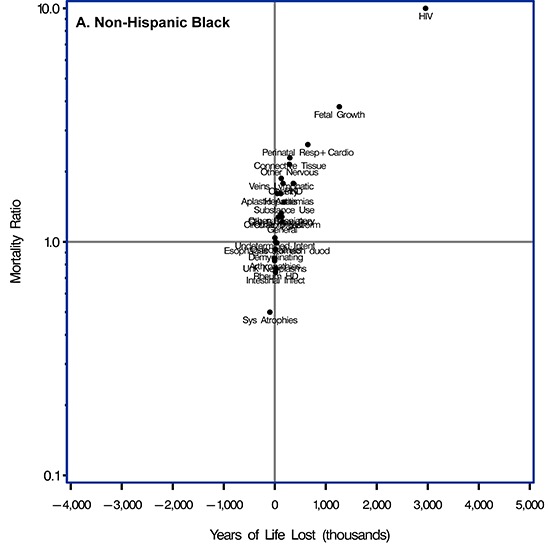

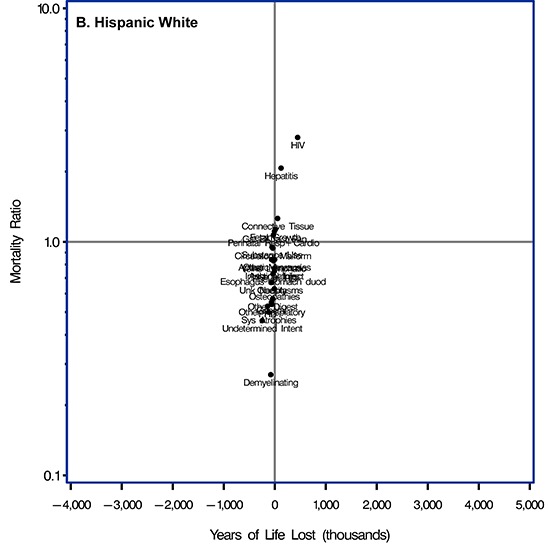

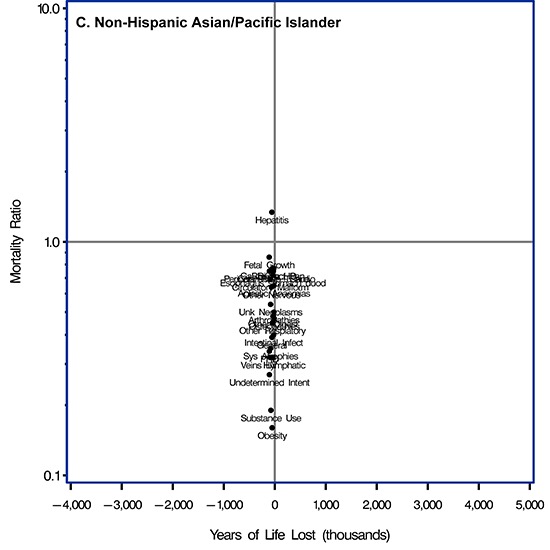

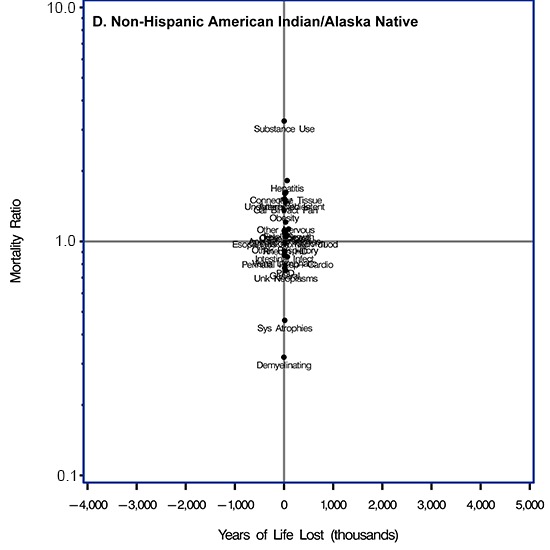
Scatterplots of relative disparities (age-adjusted mortality ratio) and absolute disparities (years of life lost) for each of 4 racial/ethnic minority groups compared with the non-Hispanic white population for the 26th- to 50^th^-ranked leading causes of death in the United States, 1999–2010. Causes of death were categorized according to the *International Classification of Diseases, 10^th^ Revision*. Scatterplot A is for non-Hispanic blacks; B is for Hispanic whites; C is for non-Hispanic Asians/Pacific Islanders; and D is for non-Hispanic American Indians/Alaska Natives. Abbreviations for categories of causes of death were used to provide clearer plots. Abbreviations: PHD, pulmonary heart disease and diseases of pulmonary circulation; General, general symptoms and signs; Unk Neoplasms, neoplasms of uncertain or unknown behavior; HIV, human immunodeficiency virus disease; Substance Use, mental or behavioral disorders due to psychoactive substance use; Gal BilTract Pan, disorders of gallbladder, biliary tract, or pancreas; Other Digest, other diseases of the digestive system; Other Respiratory, other diseases of the respiratory system; Other Nervous, other disorders of the nervous system; Sys Atrophies, systemic atrophies primarily affecting the central nervous system; Esophagus stomach duod, diseases of esophagus, stomach, or duodenum; Hepatitis, viral hepatitis; Intestinal Infect, intestinal infectious diseases; Undetermined Intent, event of undetermined intent; Fetal Growth, disorders related to length of gestation or fetal growth; Connective Tissue, systemic connective tissue disorders; Obesity, obesity or other hyperalimentation; Arthropathies, arthropathies; Veins Lymphatic, diseases of veins, lymphatic vessels, or lymph nodes, not elsewhere classified; Circulatory Malform, congenital malformations of the circulatory system.

## Supplemental Table 1. Data for Figure on Age-Adjusted Mortality Ratio (AAMR) and Difference in Years of Life Lost (dYLL), by Racial/Ethnic Group^a^ for the 25 Leading Causes of Death as Classified by the ICD-10, United States, 1999–2010^b^

**Table:**

ICD-10 Subchapter Group	Non-Hispanic Black ^a^		Hispanic White		Non-Hispanic American Indian or Alaskan Native		Non-Hispanic Asian or Pacific Islander	
	AAMR	dYLL	AAMR	dYLL	AAMR	dYLL	AAMR	dYLL
C00–C97: Malignant neoplasms	1.22	3,781,343	0.70	−3,724,049	0.80	−158,197	0.60	−1,979,722
I20–I25: Ischemic heart diseases	1.19	2,725,283	0.84	−1,728,080	0.84	−11,696	0.57	−1,368,421
I30–I51: Other forms of heart disease	1.27	2,052,395	0.59	−814,436	0.83	14,032	0.47	−500,768
I60–I69: Cerebrovascular diseases	1.44	2,000,212	0.84	−83,787	0.89	12,653	0.87	−30,913
J40–J47: Chronic lower respiratory diseases	0.65	−379,109	0.45	−1,091,025	0.82	−14,047	0.34	−624,294
E10–E14: Diabetes mellitus	2.23	1,804,677	1.65	352,592	2.37	117,812	0.82	−178,102
G30–G31: Other degenerative diseases of the nervous system	0.78	−147,356	0.69	−172,533	0.62	−11,371	0.36	−154,368
W00–X59: Other external causes of accidental injury	0.90	−84,254	0.69	−1,389,452	1.43	128,626	0.38	−917,067
F01–F09: Organic, including symptomatic, mental disorders	1.02	56,571	0.66	−146,607	0.71	−7,328	0.48	−107,692
J09–J18: Influenza and pneumonia	1.12	415,726	0.92	−78,038	1.24	33,886	0.84	−94,824
I10–I15: Hypertensive diseases	2.99	2,349,699	1.11	25,013	0.98	9,194	0.92	−72,527
V01–V99: Transport accidents	0.96	−190,089	0.94	−548,674	2.10	248,013	0.47	−724,126
N17–N19: Renal failure	2.35	1,228,893	1.04	47,097	1.40	28,364	0.68	−73,046
K70–K76: Diseases of liver	0.99	90,599	1.65	421,214	3.06	218,017	0.42	−330,094
A30–A49: Other bacterial diseases	2.20	977,585	0.88	−82,001	1.31	27,764	0.53	−122,342
I70–I78: Diseases of arteries, arterioles and capillaries	1.10	182,329	0.63	−160,671	0.80	−3,370	0.51	−94,127
X60–X84: Intentional self–harm	0.41	−1,242,755	0.46	−1,494,701	1.07	58,100	0.43	−543,803
K55–K63: Other diseases of intestines	1.15	169,834	0.75	−72,454	1.15	8,210	0.42	−75,055
G20–G25: Extrapyramidal and movement disorders	0.43	−123,259	0.66	−64,464	0.69	−2,955	0.52	−40,962
J60–J70: Lung diseases due to external agents	1.05	51,176	0.63	−82,442	0.96	1,532	0.53	−52,448
X85–Y09: Assault	7.56	4,506,949	2.64	1,249,884	3.25	97,797	0.90	−25,887
R95–R99: Ill–defined and unknown causes of mortality	1.50	787,630	0.70	−405,927	1.30	40,471	0.35	−214,763
J80–J84: Other respiratory diseases principally affecting the interstitium	0.74	−43,617	1.14	9,302	1.63	13,704	0.66	−47,064
E70–E88: Metabolic disorders	1.19	44,249	0.64	−180,021	0.88	−814	0.51	−81,486
N30–N39: Other diseases of urinary system	1.42	96,243	0.81	−24,589	1.05	2,703	0.52	−29,071

Abbreviations: ICD-10, *International Classification of Diseases, 10th Revision.*

^a^ Because the number of deaths was too small to provide stable estimates, our racial/ethnic classification excluded a small number of Hispanic American Indians/Alaska Natives, Hispanic Asians/Pacific Islanders, and Hispanic blacks from the analyses (collectively 4,072,827 of 3,530,708,204 [0.1%] of the total person-years at risk).

^b^ Each group was compared with non-Hispanic whites.

## Supplemental Table 2. Data for Supplemental Figure on Age-Adjusted Mortality Ratio (AAMR) and Difference in Years of Life Lost (dYLL), Plus Age-Adjusted Mortality Rates, by Racial/Ethnic Group^a^, for the 26th- to 50th-Ranked Causes of Death as Classified by the ICD-10, United States, 1999–2010^b^

**Table:**

ICD-10 Subchapter Group	Non-Hispanic White	Non-Hispanic Black			Hispanic White			Non-Hispanic American Indian or Alaskan Native			Non-Hispanic Asian or Pacific Islander		
	Rate	AAMR	Rate	dYLL	AAMR	Rate	dYLL	AAMR	Rate	dYLL	AAMR	Rate	dYLL
A00-A09: Intestinal infectious diseases	1.81	1.34	0.74	14,275	1.40	0.77	−1,722	1.64	0.91	1,569	0.72	0.40	−15,603
B15-B19: Viral hepatitis	1.77	2.85	1.61	118,894	3.65	2.07	139,425	3.21	1.82	13,044	2.37	1.34	−7,360
B20-B24: Human immunodeficiency virus [HIV] disease	1.80	19.43	10.81	2,957,106	5.04	2.80	475,611	2.88	1.60	12,361	0.57	0.32	−86,854
D37-D48: Neoplasms of uncertain or unknown behavior	4.36	3.61	0.83	−2,217	2.92	0.67	−54,049	3.29	0.75	−3,251	2.37	0.54	−43,205
D60-D64: Aplastic and other anemias	1.12	1.81	1.61	51,909	0.94	0.84	1,019	1.21	1.08	1,030	0.73	0.65	−4,250
E65-E68: Obesity and other hyperalimentation	1.36	2.43	1.78	162,285	0.91	0.67	−44,564	1.85	1.36	5,779	0.22	0.16	−55,233
F10-F19: Mental and behavioral disorders due to psychoactive substance use	3.03	4.49	1.48	182,083	2.87	0.95	−18,684	9.88	3.27	66,826	0.57	0.19	−140,138
G10-G12: Systemic atrophies primarily affecting the central nervous system	2.61	1.30	0.50	−95,134	1.30	0.50	−101,981	1.20	0.46	−8,240	0.92	0.35	−59,034
G35-G37: Demyelinating diseases of the central nervous system	1.26	1.16	0.93	17,085	0.34	0.27	−69,460	0.40	0.32	−5,135	0.12	0.09	−45,209
G90-G98: Other disorders of the nervous system	2.42	5.19	2.15	285,330	2.04	0.84	−42,216	2.93	1.21	4,760	1.54	0.64	−39,268
I05-I09: Chronic rheumatic heart diseases	1.14	0.88	0.77	17,729	0.86	0.75	−2,497	1.12	0.98	1,644	0.88	0.77	−160
I26-I28: Pulmonary heart disease and diseases of pulmonary circulation	4.32	7.68	1.78	364,562	2.30	0.53	−117,555	3.39	0.78	−617	1.46	0.34	−82,762
I80-I89: Diseases of veins, lymphatic vessels and lymph nodes, not elsewhere classified	1.22	2.28	1.87	127,804	1.02	0.83	−18,277	1.06	0.87	−104	0.39	0.32	−27,836
J96-J98: Other diseases of the respiratory system	2.67	3.56	1.33	128,686	1.44	0.54	−47,483	2.64	0.99	1,280	1.20	0.45	−32,465
K20-K31: Diseases of esophagus, stomach and duodenum	2.25	2.23	0.99	39,723	1.63	0.73	−26,776	2.36	1.05	1,944	1.62	0.72	−20,335
K80-K86: Disorders of gallbladder, biliary tract and pancreas	2.59	3.38	1.31	111,881	2.86	1.11	4,194	3.80	1.47	6,930	1.99	0.77	−25,654
K90-K92: Other diseases of the digestive system	2.67	3.42	1.28	74,729	1.51	0.57	−34,489	3.00	1.12	3,574	1.29	0.48	−26,618
M00-M25: Arthropathies	1.44	1.22	0.85	1,440	1.11	0.77	−5,434	2.19	1.52	3,802	0.71	0.50	−12,552
M30-M35: Systemic connective tissue disorders	1.21	2.78	2.29	295,692	1.52	1.26	63,508	1.96	1.62	7,132	0.91	0.75	1,982
M80-M94: Osteopathies and chondropathies	1.07	1.07	1.00	15,003	0.67	0.63	−6,579	1.19	1.11	994	0.51	0.47	−8,388
P05-P08: Disorders related to length of gestation and fetal growth	1.12	4.24	3.79	1,265,783	1.26	1.13	82,276	1.26	1.13	3,970	0.96	0.86	−20,805
P20-P29: Respiratory and cardiovascular disorders specific to the perinatal period	0.99	2.59	2.61	648,133	1.06	1.07	37,762	0.85	0.86	−3,830	0.74	0.75	−32,546
Q20-Q28: Congenital malformations of the circulatory system	1.27	1.63	1.28	138,912	1.20	0.94	10,650	1.34	1.06	4,250	0.88	0.69	−32,939
R50-R68: General symptoms and signs	4.31	5.26	1.22	147,760	2.35	0.55	−53,582	3.31	0.77	1,717	1.68	0.39	−44,484
Y10-Y34: Event of undetermined intent	1.78	1.86	1.04	2,761	0.82	0.46	−209,729	2.72	1.52	12,466	0.49	0.27	−107,190

Abbreviations: ICD-10, *International Classification of Diseases, 10th Revision.*

^a^ Because the number of deaths was too small to provide stable estimates, our racial/ethnic classification excluded a small number of Hispanic American Indians/Alaska Natives, Hispanic Asians/Pacific Islanders, and Hispanic blacks from the analyses (collectively 4,072,827 of 3,530,708,204 [0.1%] of the total person-years at risk).

^b^ Each group was compared with non-Hispanic whites.
